# Key Parameter Extraction for Fiber Brillouin Distributed Sensors Based on the Exact Model

**DOI:** 10.3390/s18082419

**Published:** 2018-07-25

**Authors:** Zhiniu Xu, Lijuan Zhao

**Affiliations:** School of Electrical and Electronic Engineering, North China Electric Power University, Baoding 071003, China; wzcnjxx@sohu.com

**Keywords:** fiber distributed sensors, Brillouin gain spectrum, key parameter extraction, Voigt profile, Gauss-Hermite quadrature, convergence criteria

## Abstract

Errors in the extracted key parameters directly influence the errors in the temperature and strain measured by fiber Brillouin distributed sensors. Existing key parameter extraction algorithms for Brillouin gain spectra are mainly based on simplified models, therefore, the extracted parameters may have significant errors. To ensure high accuracy in the extracted key parameters in different cases, and consequently to measure temperature and strain with high accuracy, a key parameter extraction algorithm based on the exact Voigt profile is proposed. The objective function is proposed using the least-squares method. The Levenberg-Marquardt algorithm is used to minimize the objective function and consequently extract the key parameters. The optimization process is presented in detail, at the same time the initial values obtainment method and the convergence criterion are given. The influences of the number of sample points in Gauss-Hermite quadrature on the accuracy and the computation time of the algorithm are investigated and a suggestion about the selection of the number of sample points is given. The direct algorithm, the random algorithm and the proposed algorithm are implemented in Matlab and are used to extract key parameters for abundant numerically generated and measured Brillouin gain spectral signals. The results reveal that the direct algorithm requires less computation time, but its errors are considerably larger than that of the proposed algorithm. The convergence rate of the random algorithm is about 80~90%. The proposed algorithm can converge in all cases. Even for the convergence cases, the computation time and the fitting error of the random algorithm are 1~2 times larger than those of the proposed algorithm.

## 1. Introduction

Changes in fiber temperature or strain will alter the corresponding peak values of gain, frequency shift and line width (full width at half maximum, FWHM) of Brillouin gain spectra. The temperature or strain along the fiber can be measured by analysis of the Brillouin gain spectrum, therefore, fiber distributed sensing based on Brillouin scattering is extensively used in diverse industrial and scientific fields [1,2,3,4,5,6]. The most intensively investigated topics with regard to the fiber distributed temperature and strain measurement based on Brillouin scattering include how to improve the accuracy, spatial resolution and widen the measurement range. There are hardware-based methods [7,8,9,10] and software-based methods [11,12,13]. This work is concerned with improvement of the sensing performance by use of data processing. The accuracy in the extracted Brillouin frequency shift, line width and peak value of gain directly influences the accuracy of the measured temperature and strain. Machine learning techniques have been well demonstrated and have better performance [11,12]. Additionally, the cross-correlation technique employs an elegant and exciting approach to extract the Brillouin frequency shift [13]. Its accuracy is quite similar to that of the fitting algorithms. Its paramount advantage is its extremely low computational burden. However, the line width cannot be extracted by this technique. In summary, the fitting algorithms are mainstream ones and the principal objective of this work is to improve the performance of the fitting ones, therefore, fitting algorithms are mainly reviewed below.

The parameters of the incident light have an important effect on the Brillouin gain spectrum. If the pulse width of the incident light is significantly greater than 10 ns, the Brillouin gain spectrum has a Lorentzian spectral shape [14,15]. To improve the spatial resolution, a narrower pulse will be used and the spectrum will close to a Gaussian spectral shape [16] because of the Doppler broadening. Generally, the Brillouin gain spectrum is considered as a Voigt spectral shape [17,18,19]. Because the Lorentzian and Gaussian profiles are algebraic equations, key parameter extraction algorithms based on the Lorentzian or Gaussian profiles are easy to implement and the relative algorithm studies have now reached a certain maturity [20,21]. If the profiles are centered, the Voigt profile is a line profile resulting from the convolution of a Gaussian profile and a Lorentzian profile [18]. It is not an algebraic equation. The convolution operation is relatively slower to compute than the numerical generation of the Gaussian and Lorentzian profiles, not to mention fitting a Voigt profile to the measured Brillouin gain spectrum to extract key parameters. To avoid the computational expense of the convolution operation, the Voigt profile is often approximated by a pseudo-Voigt profile which is a linear combination of a Gaussian profile and a Lorentzian profile [22,23]. Similar to the Gaussian and Lorentzian profiles, the pseudo-Voigt profile is an algebraic equation. Therefore, the key parameter extraction algorithm based on the pseudo-Voigt profile is computationally easier to implement and is nowadays the mainstream algorithm for key parameter extraction [22,23,24]. However, the above Lorentzian, Gaussian and pseudo-Voigt profiles are all simplified models of the Brillouin gain spectrum, therefore the algorithms based on them inevitably introduce errors. To improve the adaptability of the key parameter extraction algorithm, in this work, the Voigt profile rather than the other simplified one is used to approximate the Brillouin gain spectrum. Accordingly, the optimization algorithm, the initial values obtainment method and the convergence criterion for the exact model must be investigated. Related works are scarce. Reference [17] approximated the Brillouin gain spectrum along a 36 km long-range optical fiber by use of the Voigt profile to extract the Brillouin frequency shift. However, no technical details about the fitting algorithm were presented. According to the features of the Voigt profile, the line width of the corresponding Lorentzian profile can be estimated from the 20-dB spectrum width [19]. Then the line width of the corresponding Guassian profile is readily estimated from the relationship among line widths of the Lorentzian, Guassian and Voigt profiles. This algorithm may reduce the total computation time. However, many approximation formulas are used in the algorithm, therefore, significant errors may be introduced in some situations. To sum up, to date, there is still a lack of an effective algorithm to extract the key parameters from the Brillouin gain spectrum. This topic needs to be studied further.

To fix the above problems, the errors in the key parameters extracted by the algorithms based on different models are compared and the necessity to use the Voigt profile is confirmed. On the basis of such analysis, the objective function of the key parameters extraction algorithm based on the Voigt profile is determined by use of the least-squares method. The objective function is minimized using the Levenberg-Marquardt algorithm and consequently the key parameters are extracted. The initial values obtainment method and the convergence criterion are presented. The influence of the number of sample points in Gauss-Hermite quadrature on the accuracy and computation time of the algorithm is investigated and suggestions about the selection of the number of sample points are given. Based on the abovementioned investigations, a key parameters extraction algorithm based on the exact Voigt profile is proposed. The proposed algorithm is validated by extracting the key parameters for numerous numerically generated and measured Brillouin gain spectra.

## 2. Adaptability of Different Models for Key Parameters Extraction

### 2.1. Voigt Profile

If the profiles are centered, the Voigt profile is a line profile resulting from the convolution of a Gaussian profile and a Lorentzian profile [25] which can be expressed by:

(1) $$g_{B}\left(\nu \right)\;=\;A\frac{2\ln2}{\pi^{3/2}}\frac{{\Delta \nu }_{\mathrm{BL}}}{{\Delta \nu }_{\mathrm{BG}}^{2}}\int_{-\infty }^{+\infty }\frac{e^{{-x}^{2}}}{{\left[\sqrt{\ln2}\frac{\Delta \nu_{\mathrm{BL}}}{\Delta \nu_{\mathrm{BG}}}\right]}^{2}+\;{\left[2\sqrt{\ln2}\frac{{\nu \;-\;\nu }_{B}}{{\Delta \nu }_{\mathrm{BG}}}\;-\;x\right]}^{2}}dx\;$$

where *g*_B_ is the Brillouin gain; *v* is the frequency; *v*_B_ is the Brillouin frequency shift; Δ*v*_BL_ and Δ*v*_BG_ respectively are the line widths of the Lorentzian and Gaussian profiles [17]. Assume that Δ*v*_B_ is the line width of Brillouin gain spectrum; *g*_BM_ is the maximum value of Brillouin gain*.*

### 2.2. Adaptability of Different Models

Before development of the key parameter extraction algorithm based on the Voigt profile, we should check the adaptability of the existing different models. Without loss of generality, *v*_B_ = 10.7 GHz, *A* = 0.2, Δ*v*_BL_ = 0.01 GHz. Δ*v*_BG_ ranges from 0.01 GHz to 0.15 GHz. Fifteen sets of Brillouin gain spectra are numerically generated according to Equation (1). To avoid the errors caused by not having enough sample points in Gauss-Hermite quadrature, the number of sample points is set to 1000. Three key parameters extraction algorithms based on Lorentzian profile [14], Gaussian profile [16] and pseudo-Voigt profile models [22], respectively, are implemented in Matlab, and the statistical results of the relative errors in *g*_BM_, *v*_B_ and Δ*v*_B_ extracted by the different model-based algorithms are presented in Table 1. *E*_max_ means the maximum value of the error. *E*_mean_ means the mean value of the error magnitude. For example, the second column of Table 1 are the maximum errors in *g*_BM_, *v*_B_ and Δ*v*_B_ extracted by the Lorentzian profile.

From Table 1 it can be seen that *v*_B_ extracted by the three models contains no error in different cases. However, the three models introduce varying degrees of errors in the extracted *g*_BM_ and Δ*v*_B_. The maximum errors in *g*_BM_ extracted by the three models are up to 7.55%, −4.33% and 1.51%, respectively. The maximum errors in the extracted Δ*v*_B_ are −14.85%, 10.43% and −1.76%, respectively. The above results reveal that the key parameters extracted by the extensively used three simplified model (the Lorentzian profile, Gaussian profile and pseudo-Voigt profile)-based algorithms, in theory, may contain significant errors. The exact model must be used. Therefore, the key parameter extraction algorithm based on the exact model (Voigt profile) needs to be urgently studied, which is the core part of this work.

## 3. Key Parameters Extraction Algorithm Based on Voigt Profile

### 3.1. Objective Function

Because Equation (1) does not have an analytical solution, it must be numerically solved. Gauss-Hermite quadrature [26] is particularly suitable for approximating the value of integrals containing $e^{-x^{2}}$, therefore, it is used to calculate Equation (1). The Gauss-Hermite quadrature with number of sample points of *M* can be expressed as follows:

(2) $$\int_{-\infty }^{+\infty }e^{{-x}^{2}}f\left(x\right)dx\;\approx \;\overset{M}{\underset{m\;=\;1}{\sum }}w_{m}{f\;(x}_{m})\;$$

where *x_m_* is the root of the physicists’ version of the Hermite polynomial *H_M_*(*x*) with an order of *M*, (*m* = 1, 2, ..., *M*) and the associated weight *w_m_* is given by Equation (3) [27]:

(3) $$\;w_{m}\;=\;\frac{2^{M\;-\;1}M\text{!}\sqrt{\pi }}{M^{2}{{[H}_{M\;-\;1}{(x}_{m})]}^{2}}\;$$

According to Equation (2), Equation (1) can be rearranged as:

(4) $$\;g_{B}\left(\nu \right)\;\approx \;A\frac{2\ln2}{\pi^{3/2}}\frac{{\Delta \nu }_{\mathrm{BL}}}{{\Delta \nu }_{\mathrm{BG}}^{2}}\overset{M}{\underset{m\;=\;1}{\sum }}w_{m}\frac{1}{{\left[\sqrt{\ln2}\frac{\Delta \nu_{\mathrm{BL}}}{\Delta \nu_{\mathrm{BG}}}\right]}^{2}+\;{\left[2\sqrt{\ln2}\frac{{\nu \;-\;\nu }_{B}}{{\Delta \nu }_{\mathrm{BG}}}\;-\;x_{m}\right]}^{2}}\;$$

Assume that *v_i_* and *g*_B*i*_ respectively are the *i*^th^ scanning frequency and the corresponding Brillouin gain, where *i* = 0, 1, 2, …, *N* − 1, *N* is the number of frequency scanning. Then the objective function determined by use of the least-squares method as follows:

(5) $$\;E=\overset{N\;-\;1}{\underset{i\;=\;0}{\sum }}e_{i}^{2}\;$$

where *E* is the sum of the squared normal distances between the measured or numerically generated profile coordinates and the expected profile:

(6) $$e_{i}{\;=\;g}_{B}\left(\nu_{i}\right){\;-\;g}_{Bi}\;=\;A\frac{2\ln2}{\pi^{3/2}}\frac{{\Delta \nu }_{\mathrm{BL}}}{{\Delta \nu }_{\mathrm{BG}}^{2}}\sum_{m\;=\;1}^{M}w_{m}\frac{1}{{\left[\sqrt{\ln2}\frac{\Delta \nu_{\mathrm{BL}}}{\Delta \nu_{\mathrm{BG}}}\right]}^{2}+\;{\left[2\sqrt{\ln2}\frac{\nu_{i}{-\nu }_{B}}{{\Delta \nu }_{\mathrm{BG}}}{\;-\;x}_{m}\right]}^{2}}{-\;g}_{Bi},\;i\;=\;0,\;1,\;2,\;\ldots ,\;N\;-\;1$$

### 3.2. Optimization Algorithm

The above objective function belongs to a nonlinear least-squares problem. The Levenberg-Marquardt algorithm is most appropriate for minimization of the nonlinear least-squares problem. Therefore, it is chosen and the variables can be updated by:

(7) $$W\left(l+1\right)=W\left(l\right)-{\left(J{\left(l\right)}^{T}J\left(l\right)+\lambda I\right)}^{-1}J{\left(l\right)}^{T}e\left(l\right)$$

where ***e*** = [*e*_0_, *e*_1_, …, *e_N_*_−1_]^T^ is the error vector, and ***W*** = [*W*_1_, *W*_2_, *W*_3_, *W*_4_]^T^ = [*A*, *v*_B_, Δ*v*_BL_, Δ*v*_BG_]^T^ is the variable vector. **I** is a 4 × 4 unit matrix. *l* is the iteration number. Superscript T means transposition. When a step increases *E*, *λ* is multiplied by 10. At the same time, the change in ***W*** is disregarded, and the previous values of ***W*** are retained. *λ* is divided by 10 whenever a step would result in a decreased *E*. The initial value of *λ* is set to 1.

***J*** is a *N ×* 4 Jacobian matrix, $J_{ij}=\partial e_{i}/\partial W_{j}$, 0 ≤ *i* ≤ *N* − 1, 1 ≤ *j* ≤ 4, is an element of the Jacobian matrix which is represented as follows:

(8) $$J_{i1}\;=\frac{\partial e_{i}}{\partial A}\;=\;\frac{2\ln2}{\pi^{3/2}}\frac{{\Delta \nu }_{\mathrm{BL}}}{{\Delta \nu }_{\mathrm{BG}}^{2}}\overset{M}{\underset{m\;=\;1}{\sum }}w_{m}\frac{1}{{\left[\sqrt{\ln2}\frac{\Delta \nu_{\mathrm{BL}}}{\Delta \nu_{\mathrm{BG}}}\right]}^{2}+\;{\left[2\sqrt{\ln2}\frac{\nu_{i}{\;-\;\nu }_{B}}{{\Delta \nu }_{\mathrm{BG}}}{\;-\;x}_{m}\right]}^{2}}.\;$$

(9) $$\;J_{i2}\;=\frac{\partial e_{i}}{\partial \nu_{B}}\;=\;A\frac{8{\left(\sqrt{\ln2}\right)}^{3}}{\pi^{3/2}}\frac{{\Delta \nu }_{\mathrm{BL}}}{{\Delta \nu }_{\mathrm{BG}}^{3}}\overset{M}{\underset{m\;=\;1}{\sum }}w_{m}\frac{\left[2\sqrt{\ln2}\frac{\nu_{i}{-\nu }_{B}}{{\Delta \nu }_{\mathrm{BG}}}{-x}_{m}\right]}{{\left\{{\left[\sqrt{\ln2}\frac{\Delta \nu_{\mathrm{BL}}}{\Delta \nu_{\mathrm{BG}}}\right]}^{2}+{\left[2\sqrt{\ln2}\frac{\nu_{i}{-\;\nu }_{B}}{{\Delta \nu }_{\mathrm{BG}}}{\;-\;x}_{m}\right]}^{2}\right\}}^{2}}\;$$

(10) $$\begin{array}{c} J_{i3\;}=\;\frac{\partial e_{i}}{\partial \Delta \nu_{\mathrm{BL}}}\;=\;A\frac{2\ln2}{\pi^{3/2}}\frac{1}{{\Delta \nu }_{\mathrm{BG}}^{2}}\overset{M}{\underset{m\;=\;1}{\sum }}w_{m}\frac{1}{{\left[\sqrt{\ln2}\frac{\Delta \nu_{\mathrm{BL}}}{\Delta \nu_{\mathrm{BG}}}\right]}^{2}+\;{\left[2\sqrt{\ln2}\frac{\nu_{i}{\;-\;\nu }_{B}}{{\Delta \nu }_{\mathrm{BG}}}{\;-\;x}_{m}\right]}^{2}}\\ \;\;\;\;\;\;\;\;\;\;\;\;\;\;\;\;\;\;\;\;\;\;\;\;\;\;\;\;\;\;\;\;\;\;\;\;\;\;\;-A\frac{{4\ln}^{2}2}{\pi^{3/2}}\frac{{\Delta \nu }_{\mathrm{BL}}^{2}}{{\Delta \nu }_{\mathrm{BG}}^{4}}\overset{M}{\underset{m\;=\;1}{\sum }}w_{m}\frac{1}{{\left\{{\left[\sqrt{\ln2}\frac{\Delta \nu_{\mathrm{BL}}}{\Delta \nu_{\mathrm{BG}}}\right]}^{2}+{\left[2\sqrt{\ln2}\frac{\nu_{i}{\;-\;\nu }_{B}}{{\Delta \nu }_{\mathrm{BG}}}{\;-\;x}_{m}\right]}^{2}\right\}}^{2}} \end{array}$$

(11) $$\begin{array}{c} J_{i4}\;=\;\frac{\partial e_{i}}{\partial \nu_{\mathrm{BG}}}\;=\;A\frac{-\;4\ln2}{\pi^{3/2}}\frac{\Delta \nu_{\mathrm{BL}}}{{\Delta \nu }_{\mathrm{BG}}^{3}}\overset{M}{\underset{m\;=\;1}{\sum }}w_{m}\frac{1}{{\left[\sqrt{\ln2}\frac{\Delta \nu_{\mathrm{BL}}}{\Delta \nu_{\mathrm{BG}}}\right]}^{2}+\;{\left[2\sqrt{\ln2}\frac{\nu_{i}{-\nu }_{B}}{{\Delta \nu }_{\mathrm{BG}}}{\;-\;x}_{m}\right]}^{2}}\\ \;\;\;\;\;\;\;\;\;\;\;\;\;\;\;\;\;\;\;\;\;\;\;\;\;\;\;\;\;\;\;\;\;\;\;\;\;\;\;+A\frac{2\ln2}{\pi^{3/2}}\frac{\Delta \nu_{\mathrm{BL}}}{\Delta_{\nu_{\mathrm{BG}}}^{2}}\overset{M}{\underset{m=1}{\sum }}w_{m}\frac{2\ln2\frac{\Delta_{\nu_{\mathrm{BL}}}^{2}}{\Delta_{\nu_{\mathrm{BG}}}^{3}}+4\sqrt{\ln2}\frac{\nu_{i}-\nu_{B}}{\Delta_{\nu_{\mathrm{BG}}}^{2}}\left[2\sqrt{\ln2}\frac{\nu_{i}-\nu_{B}}{\Delta \nu_{\mathrm{BG}}}-x_{m}\right]}{{\left\{{\left[\sqrt{\ln2}\frac{\Delta \nu_{\mathrm{BL}}}{\Delta \nu_{\mathrm{BG}}}\right]}^{2}+{\left[2\sqrt{\ln2}\frac{\nu_{i}-\nu_{B}}{\Delta \nu_{\mathrm{BG}}}-x_{m}\right]}^{2}\right\}}^{2}} \end{array}$$

### 3.3. Initial Values Obtainment

The initial guesses of *A*, *v*_B_, Δ*v*_BL_ and Δ*v*_BG_ have a big influence on the rate of the objective function optimization. If the initial guesses are close to a local maxima, the Levenberg-Marquardt algorithm may converge to the local maxima and significant errors will be introduced. Therefore, a fast and accurate method of obtaining the initial values is needed.

For the Voigt profile, Δ*v*_B_ can be found from the widths of the associated Gaussian and Lorentzian widths. A better approximation [28] is given by:

(12) $$\;\Delta \nu_{B}\approx 0.5346\Delta \nu_{\mathrm{BL}}+\sqrt{0.2166{\Delta \nu }_{\mathrm{BL}}^{2}+{\Delta \nu }_{\mathrm{BG}}^{2}}\;$$

Since we don’t know the values of Δ*v*_BL_ and Δ*v*_BG_ in advance, let us assume that Δ*v*_BL_ = Δ*v*_BG_, thus the initial guesses of Δ*v*_BL_ and Δ*v*_BG_ can be calculated by Equation (13):

Δ*v*_BL_ = Δ*v*_BG_ = Δ*v*_B_/1.6376 (13)

*g*_B_(*v*) reaches the maximum value at *v* = *v*_B_ which can be calculated by Equation (14) [29]:

(14) $$g_{B}\left(\nu_{B}\right)=2A\sqrt{\frac{\ln2}{\pi }}\frac{1}{\Delta \nu_{\mathrm{BG}}}e^{{\left(\sqrt{\ln2}\frac{\Delta \nu_{\mathrm{BL}}}{\Delta \nu_{\mathrm{BG}}}\right)}^{2}}(1\;-\;\frac{2}{\sqrt{\pi }}\int_{0}^{\sqrt{\ln2}\frac{\Delta \nu_{\mathrm{BL}}}{\Delta \nu_{\mathrm{BG}}}}e^{{-x}^{2}}dx)\;$$

Assume that the gain in the measured spectrum reaches the maximum value when *v* = *v*_P_ and the corresponding maximum gain is *g*_BM_. According to Equation (14), the initial guesses of *v*_B_ and *A* can be calculated by Equations (15) and (16), respectively:

*v*_B_ = *v*_P_ (15)

(16) $$\;A\;=\;\frac{g_{\mathrm{BM}}}{2\sqrt{\frac{\ln2}{\pi }}\frac{1}{\Delta \nu_{\mathrm{BG}}}e^{{\left(\sqrt{\ln2}\frac{\Delta \nu_{\mathrm{BL}}}{\Delta \nu_{\mathrm{BG}}}\right)}^{2}}(1\;-\;\frac{2}{\sqrt{\pi }}\int_{0}^{\sqrt{\ln2}\frac{\Delta \nu_{\mathrm{BL}}}{\Delta \nu_{\mathrm{BG}}}}e^{{-x}^{2}}dx)}\;$$

### 3.4. Convergence Criterion

Due to the computational expense of the convolution operation in Equation (1), the key parameter extraction algorithm based on the Voigt profile requires more computation time than that of the Lorentzian profile-based one. Therefore, the computation time is the key factor in the algorithm. Although good initial guesses can reduce the computational burden, an appropriate convergence criterion can further decrease the number of iterations and the computational expense, and consequently, it is needed. After repeated tries, the result indicates that if the variation among successive iterations is less than a certain value, then the algorithm converges. Assume that ***W***(*i*) = [*W*_1_(*i*), *W*_2_(*i*), *W*_3_(*i*), *W*_4_(*i*)]^T^, the stopping condition is defined as follows:

(17) $$\;\left|\frac{W_{j}\left(i\right){-W}_{j}\left(i\;-\;1\right)}{W_{j}\left(i\;-\;1\right)}\right|\leq 10^{-5};\;i\;=\;l\;-\;4,\;l\;-\;3,\;\cdots ,\;l;\;j\;=\;1,\;2,\;3,\;4\;$$

The maximum iteration number *l*_max_ is set to 500. Certainly *l*_max_ can be adjusted according to the practical situations. Once Equation (17) is satisfied or the iteration number is not more than *l*_max_, the algorithm stops iterating. The iteration number is *l* and *W*(*l*) is taken as the final solution.

To validate the proposed convergence criterion, two other convergence criteria are introduced and are used to validate the proposed one (Equation (18)). The stopping condition corresponding to the first one can be expressed by Equation (18). In the second criterion, the iteration number is fixed at 500:

(18) $$\;\left|\frac{W_{j}\left(i\right){\;-\;W}_{j}\left(i\;-\;1\right)}{W_{j}\left(i\;-\;1\right)}\right|\;\leq {\;10}^{-5};\;i\;=\;l\;-\;2,\;l\;-\;1,\;l;\;j\;=\;1,\;2,\;3,\;4\;$$

A large amount of noise-free Brillouin gain spectra are numerically generated. According to the single-mode fiber properties, *v*_B_ is set to a random value from 10 GHz to 13 GHz and *A* is a random value from 0 to 0.3. Both Δ*v*_BL_ and Δ*v*_BG_ are random values from 0.01 GHz to 0.15 GHz. The number of sample points in Gauss-Hermite quadrature is 1000 which is the same as in Section 2.2. 10,000 sets of Brillouin gain spectra are numerically generated based on Equation (4). The frequency is scanned in the range from *v*_B_ − (Δ*v*_BL_ + Δ*v*_BG_) to *v*_B_ + (Δ*v*_BL_ + Δ*v*_BG_) and the scanned frequency interval is (Δ*v*_BL_ + Δ*v*_BG_)/20. In the key parameters extraction algorithm, the number of sample points is set to 100 which is validated in Section 4.2. *E_g_*_mean_, *E_v_*_mean_ and *E*_Δ*v*mean_ are the mean values of the error magnitude in the extracted *g*_BM_, *v*_B_ and Δ*v*_B_, respectively. *E*_M_ is the mean value of the sum of the squared normal distances between the profile coordinates and the expected profile. *T*_M_ and *l*_M_ respectively are the mean values of the computation time and the iteration number. The key parameters extraction algorithm based on the Voigt profile is used. If the iteration number is fixed at 500 (*l* = 500), the algorithm will converge in all cases. Therefore, the corresponding results are taken as the exact values. The statistical results of errors in the extracted key parameters, the sum squared error, the computation time and the iteration number corresponding to different convergence criterions are summarized in Table 2. Note however that once the correction terms of the variables are NaN (not a number), the algorithm will stop. Therefore, even the iteration number is set to 500, the real iteration number is less than 500.

As shown in Table 2, the extracted key parameters according to Equation (17) are the same as the extracted parameters with the fixed iteration number of 500 (500 is large enough). The mean value of iteration number corresponding to Equation (17) is 9.74 which is much less than 500 and 3.27 × 10^2^. At the same time, the corresponding computation time is only 2.70 × 10^−2^ s and is considerably less than 1.28 × 10^−1^ s corresponding to the fixed iteration number of 500. The mean values of the iteration number and the computation time corresponding to Equation (18) are 7.74 and 2.48 × 10^−2^ s, respectively. Although the errors in the extracted parameters and the sum squared error corresponding to Equations (17) and (18) are the same, the iteration number and the computation time of Equation (18) respectively are less than those of Equation (17). According to Table 2, it seems that Equation (18) rather than Equation (17) should be taken as the stopping condition.

**Figure 1:**
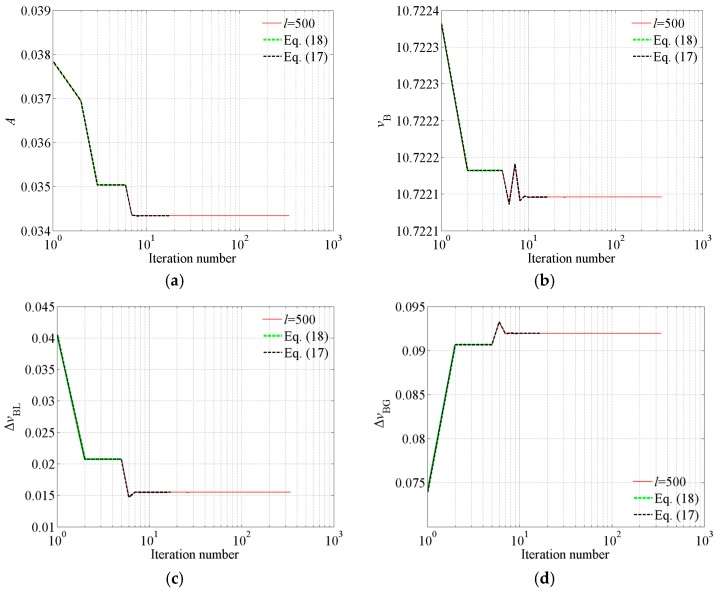
Change of the extracted key parameters corresponding to different convergence criterions with the iteration number. (**a**) *A*, (**b**) *v*_B_, (**c**) Δ*v*_BL_, (**d**) Δ*v*_BG_.

Not only noise-free Brillouin gain spectra but also noisy ones need to be investigated. The noisy Brillouin gain spectra along a single-mode 9/125 μm fiber are measured by a Brillouin optical time-domain reflectometer (BOTDR, model AV6419, China Electronics Technology Instruments Co., Ltd., Tsingtao, China). The wavelength of the incident light is 1550 nm. The pulse width is 10 ns. The average number of waveforms is 2^10^. The mean SNR is 15.18 dB. For a typical case, change of the extracted key parameters corresponding to different convergence criterions with iteration number is shown in Figure 1.

From Figure 1 we discover that the extracted parameters remain constant in four successive iterations even though the extracted key parameters are quite different from the optimal solution. However, in Equation (18), whether or not the algorithm converges is determined according to variation in the extracted parameters in successive four iterations. Therefore, Equation (18) is not reliable. Therefore, if the inappropriate convergence criterion is used, such as Equation (18), significant errors may be introduced. Generally, if the extracted parameters remain nearly constant in successive six iterations, the algorithm has converged. Therefore, in the proposed convergence criterion (Equation (17)), the variation in the extracted parameters in successive six iterations is used to judge convergence or not. At the same time, according to the convergence criterion, the unnecessary iterations are avoided. Therefore, the established convergence criterion can not only ensure converge but also corresponds to less computational burden. Therefore, Equation (17) is reliable.

### 3.5. Flowchart of the Proposed Algorithm

**Figure 2:**
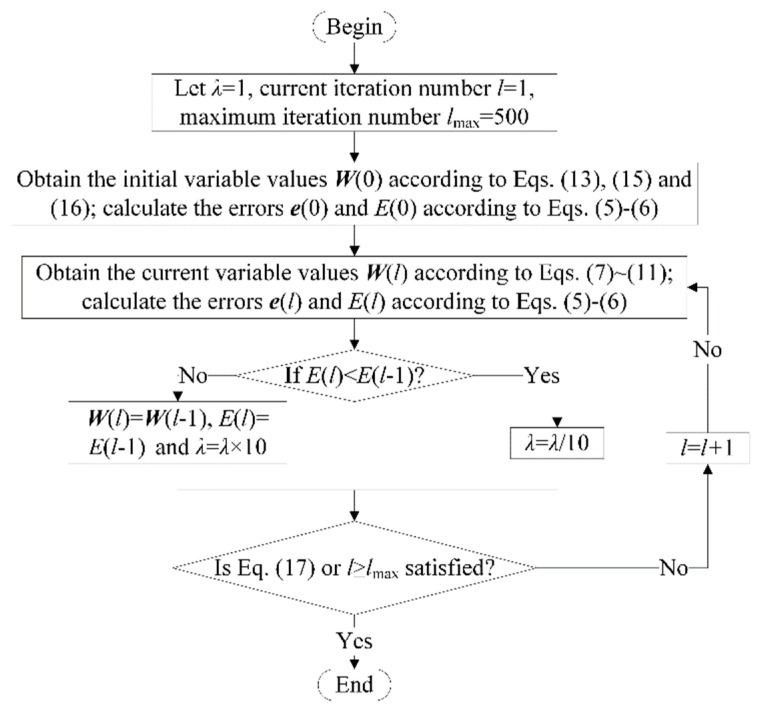
Flowchart of the proposed algorithm.

The flowchart of the proposed algorithm is illustrated in Figure 2.

## 4. Influence of Number of Sample Points in Gauss-Hermite Quadrature

The number of sample points in Gauss-Hermite quadrature has an important effect on the accuracy and computation time of the algorithm. These influences are investigated in this section.

### 4.1. Influence on Spectrum Approximation

In this section, the influence of the number of sample points on the error in the Voigt profile numerically generated according to Equation (4) (Gauss-Hermite quadrature) is investigated. The number of sample points ranges from 5 to 1000. Similar to Section 3.4, the Voigt profile with number of sample points of 1000 is considered as the exact one. The other parameters are the same as in Section 3.4. The statistical results of errors in the numerically generated Voigt waveshape with different numbers of sample points are presented in Table 3. 

**Figure 3:**
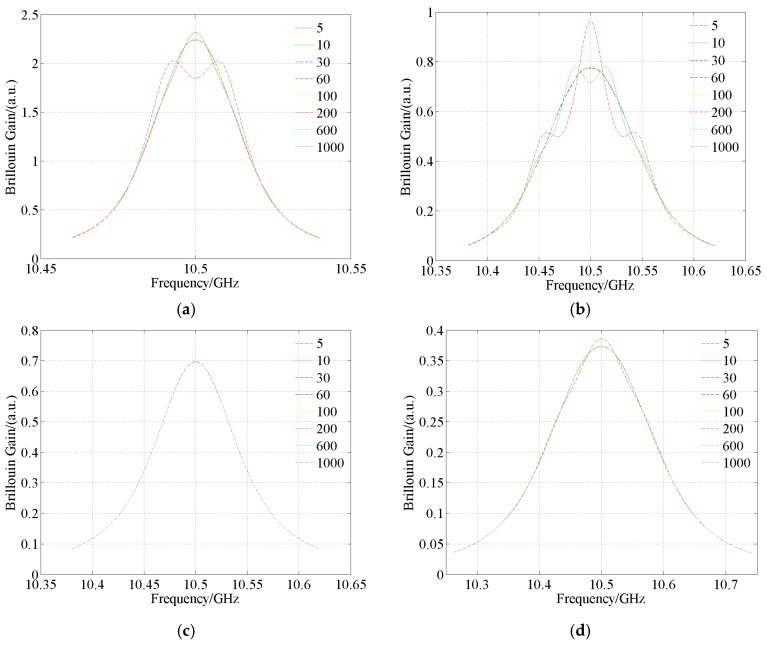
Four typical Voigt waveshapes numerically generated by Gauss-Hermite quadrature with different numbers of sample points. (**a**) Δ*v*_BL_ = 0.02 GHz, Δ*v*_BG_ = 0.02 GHz; (**b**) Δ*v*_BL_ = 0.04 GHz, Δ*v*_BG_ = 0.08 GHz; (**c**) Δ*v*_BL_ = 0.08 GHz, Δ*v*_BG_ = 0.04 GHz; (**d**) Δ*v*_BL_ = 0.12 GHz, Δ*v*_BG_ = 0.12 GHz.

Assume that *E*_1_ is the ratio of the maximum error magnitude in the Brillouin gain within the whole frequency scanning range to the maximum value of the Brillouin gain. *E*_1min_, *E*_1max_, *E*_1mean_ and *E*_1std_ respectively are the minimum, maximum, mean values and the standard deviation of *E*_1_. Let *E*_2_ is the ratio of the mean value of error magnitude in the Brillouin gain within the whole frequency scanning range to the maximum value of the Brillouin gain. *E*_2min_, *E*_2max_, *E*_2mean_ and *E*_2std_ respectively are the minimum, maximum, mean values and the standard deviation of *E*_2_. To clearly demonstrate the influence of the number of sample points on accuracy in numerically generated spectrum, four typical cases are chosen. *A* and *v*_B_ are set to 0.1 and 10.5 GHz, respectively. Δ*v*_BL_ and Δ*v*_BG_ are respectively set to 0.02 GHz and 0.02 GHz, 0.04 GHz and 0.08 GHz, 0.08 GHz and 0.04 GHz, 0.12 GHz and 0.12 GHz. Four typical Voigt waveshapes are displayed in Figure 3. To present them clearly, the scanned frequency interval in Figure 3 is set to (Δ*v*_BL_ + Δ*v*_BG_)/2000. Brillouin gain is a dimensionless parameter. The order of magnitude of Brillouin gain is of little value to the fiber distributed sensing based on Brillouin gain. Most of the related literature doesn’t care about the order of magnitude of Brillouin gain. Therefore, its real magnitude is not presented in the paper. The unit for the vertical axes in the paper is a.u. which means arbitrary unit [30,31].

As seen in Table 3 the errors in the numerically generated Voigt profile decrease with increasing number of sample points. At the same time, the decline rate also decreases with increasing number of sample points. Finally, the errors will tend to zero. *E*_1mean_ and *E*_2mean_ corresponding to number of sample points of 100 are 1.95 × 10^−3^ and 5.51 × 10^−4^, respectively. The accuracy is acceptable. Note however that the Voigt profile with number of sample points of 1000 is considered as the exact one. Therefore, no corresponding errors data are presented in Table 3.

### 4.2. Influence on the Computation Time and Accuracy of the Voigt Profile-Based Algorithm

The number of sample points is set to 1000 and the other parameters are the same as in Section 3.4. A large number of Brillouin gain spectra are numerically generated based on Equation (4). For the noise-free cases, the statistical results of errors in the key parameters extracted by the Voigt profile-based algorithm with different numbers of sample points are included in Table 4. *E_g_*_mean_, *E_v_*_mean_ and *E*_Δ*v*mean_ respectively are the mean value of the error magnitude in the extracted *g*_BM_, *v*_B_ and Δ*v*_B_. Similarly, *E_g_*_std_, *E_v_*_std_ and *E*_Δ*v*std_ are the standard deviation of the error in the extracted *g*_BM_, *v*_B_ and Δ*v*_B_, respectively.

According to Table 4, the errors in the extracted parameters decrease with increasing number of sample points. Finally, it tends to zero for the noise-free cases. The noise-free case is to simulate the case with large enough average number of waveforms. However, the noise is inevitable in the measured Brillouin gain spectrum. The SNR should not be set to a too low value. Otherwise, a too low value of the number of sample points will be selected. Then, for high SNR cases, significant errors will be introduced. Therefore, the SNR is set to 40 dB and the other parameters are the same as the noise-free cases. The statistical results of errors in the extracted key parameters are presented in Table 5.

From Table 5 we discover that the errors in the extracted key parameters decrease with increasing number of sample points. Because of noise, the rate of decrease is slower than that of the noise-free spectra in Table 4. For the noisy spectra, the errors corresponding to the number of sample points of 100 are very close to the errors corresponding to the number of sample points of 1000. *v*_B_ possesses the highest accuracy among the three parameters and is less sensitive to the number of sample points. This is beneficial for temperature and strain measurement based on Brillouin scattering. This is due to the fact that temperature and strain are measured mainly according to *v*_B_. However, to achieve simultaneous measurement of temperature and strain, Δ*v*_B_ may be needed. Δ*v*_B_ depends solely on Δ*v*_BL_ and Δ*v*_BG_. The errors of Δ*v*_BL_ and Δ*v*_BG_ decrease with increasing number of sample points. This is the main reason why too low number of sample points is inappropriate. According to Table 4 and Table 5, the proposed algorithm can converge in all cases independent of which number of sample points is used and no matter on whether or not noise-free spectra are used. The errors in the extracted key parameters are mainly caused by noise and are not large enough number of sample points. The above results validate the proposed optimization algorithm, the initial values obtainment method and the convergence criterion.

**Figure 4:**
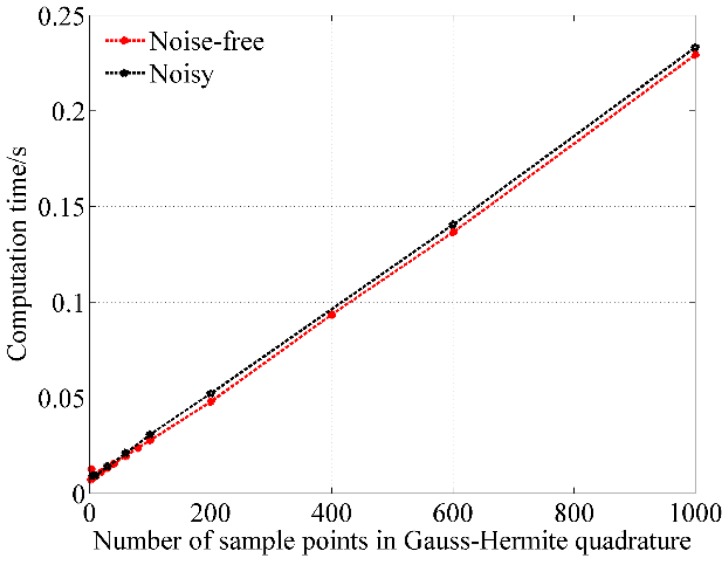
Change of the mean computation time with the number of sample points.

To further present the relationship between the number of sample points and the computation time, change of mean computation time of noisy and noise-free cases with the number of sample points are shown in Figure 4. From Figure 4 it can be seen that the computation time increases linearly with the number of sample points irrespective of noisy and noise-free cases. To improve the accuracy, more sample points should be used. To improve real-time performance, less number of sample points should be used. Selection of the number of sample points in Gauss-Hermite quadrature is thus a balance between the accuracy and the computation time. At the same time, the error increases with decreasing SNR. Therefore, less number of sample points should be selected for noisy spectra. To sum up, the number of sample points in Gauss-Hermite quadrature is generally suggested to choose 100. In comparison with the number of sample points of 1000, the selection of number of sample points of 100 can not only ensure high accuracy but also decrease computation time to about one eighth of the original value. Of course, this number can be adjusted according to the practical situations.

## 5. Validation

### 5.1. Numerically Generated Signals

**Figure 5:**
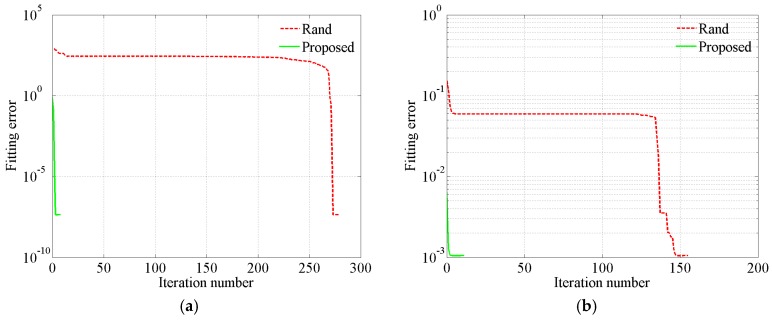
Change of the sum of the squared normal distances with iteration number. (**a**) Noise-free; (**b**) Noisy.

To compare with the proposed algorithm, the algorithm proposed by [19] (called the direct algorithm in the paper) and the random algorithm are also implemented in Matlab. In the random algorithm, the initial guesses of the variables are set to some random values within a certain range. According to single-mode fiber properties, the initial guesses of Δ*v*_BL_ and Δ*v*_BG_ are random values from 0.01 GHz to 0.15 GHz. The initial guess of *v*_B_ is a random value from 10 GHz to 13 GHz. The initial guess of *A* is a random value from 0 to 0.3. The objective function, the optimization algorithm and the convergence criterion are the same as the proposed algorithm. Equation (4) must be solved in both the numerical generation of Voigt profiles and the Voigt-profile based key parameters extraction algorithm. It means that the number of sample points in Gauss-Hermite quadrature must be determined. Similar to Section 4.2, a large number of Brillouin gain spectra are numerically generated based on Equation (4) with the number of sample points of 1000. According to Section 4.2, the number of sample points in the Voigt profile-based parameters extraction algorithm is set to 100. For the noisy signals, the SNR is set to 20 dB and the other parameters are the same as in Section 3.4. For a typical noisy and noise-free Brillouin gain spectra, change of the sum of the squared normal distances with iteration number is displayed in Figure 5. In the direct algorithm, no iteration process is needed. Therefore, no data about it is presented in Figure 5. From Figure 5, the proposed algorithm not only can ensure convergence but also requires less computation time. At the same time, the aforementioned results validate the proposed initial values obtainment method (Equations (13), (15) and (16)), objective function (Equations (5) and (6)) and convergence criterion (Equation (17)). The statistical results of errors in the key parameters extracted by different algorithms are presented in Table 6 and at the same time the computation time is included. It is noted that the direct algorithm uses the 20-dB Brillouin gain to calculate Δ*v*_BL_ which imposes a very high requirement on its frequency scanning range. Consequently, more frequency scans are needed.

From Table 6, we can see that the mean values of the error magnitude of the random algorithm are quite significant. For the noise-free cases, the errors of the random algorithm are about 10^6^~10^9^ times larger than that of the proposed one. For the noisy cases, the errors of the random algorithm are about 10~10^12^ times larger than that of the proposed one. The above results validate the proposed initial values obtainment method (Equations (13), (15) and (16)). In practice, the parameters extracted by the random algorithm do not have to contain such appreciable errors. Once converged, the parameters extracted by the random algorithm may be the same as the proposed algorithm. For 10,000 sets of noise-free and noisy Brillouin gain spectra, the convergence rates of the random algorithm are 88.16% and 77.95%, respectively. The errors in the parameters extracted by the direct algorithm are considerably larger than that of the proposed algorithm. For the noise-free cases, *v*_B_ extracted by the proposed algorithm contains no error. However, the error in *v*_B_ extracted by the direct algorithm is about 10^−3^ GHz. The errors in Δ*v*_B_ and *g*_BM_ extracted by the direct algorithm are considerably larger than those of the proposed algorithm. The errors in the parameters extracted by the three algorithms for the noisy cases are larger than that of the noise-free cases. Anyhow, the accuracy of the proposed algorithm is significantly higher than that of the other two algorithms.

Not only is the error in the extracted parameters but also the computation time of the random algorithm is larger than that of the proposed algorithm. The computation times of the random algorithm for the noise-free and noisy cases respectively are 3.86 and 2.56 times that of the proposed algorithm. This is most likely because the initial guesses provided by the random algorithm are quite different from the optimal solution. Generally, a greater number of iterations is needed. The direct algorithm requires the least computational effort among the three algorithms and its computation time is only one-third of that of the proposed algorithm. However, it has a high requirement on the scanned frequency interval and the frequency scanning range which will increase the measurement time.

### 5.2. Measured Signals

#### 5.2.1. Adaptability of Different Models

**Figure 6:**
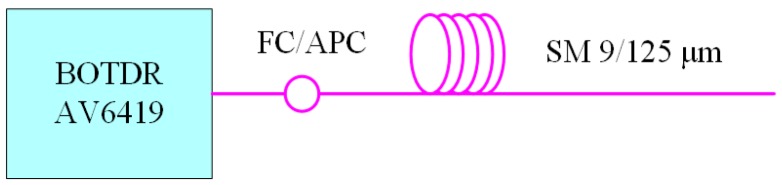
The schematic diagram of Brillouin gain spectrum measurement for a single-mode fiber.

The experimental setup is sketched in Figure 6. Brillouin gain spectra along a single-mode 9/125 μm fiber with a length of 1 km are measured by the model AV6419 BOTDR. 

**Figure 7:**
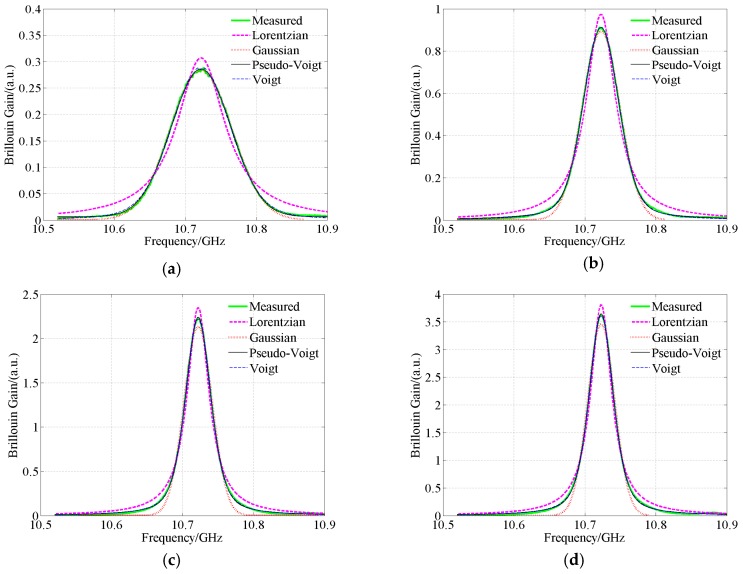

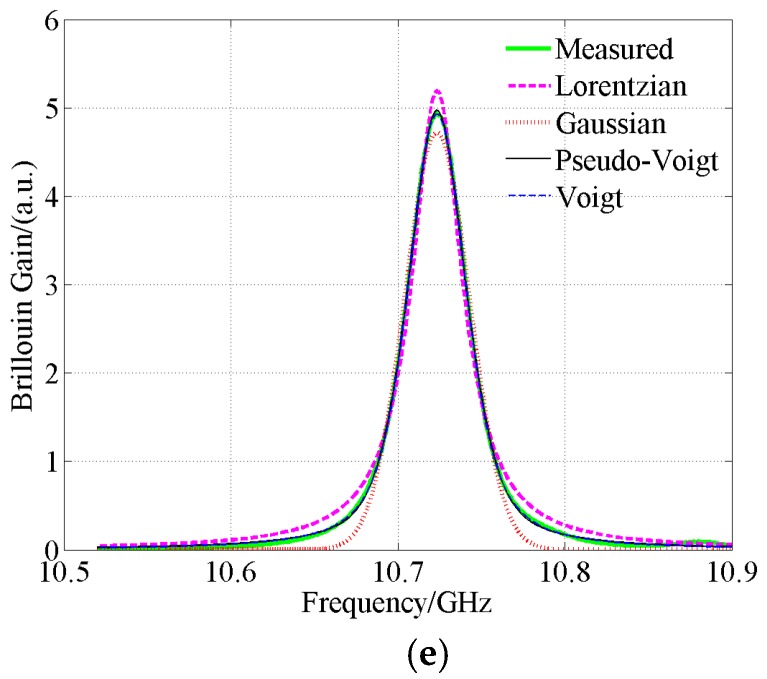
Waveshapes fitted by different models for spectra with different pulse widths, measured signals with average number of 2^18^. (**a**) 10 ns; (**b**) 20 ns; (**c**) 50 ns; (**d**) 100 ns; (**e**) 200 ns.

The sampling resolution and the frequency scanning interval are set to 0.1 m and 1 MHz, respectively. The wavelength of the incident light is 1550 nm. The pulse width is set to a value ranging from 10 ns to 200 ns. The frequency scanning ranges from 10.52 GHz to 10.92 GHz. The average number of waveforms is 2^18^. The SNR of the measured spectra ranges from 32.76 dB to 35.84 dB. These signals are spectra with high SNR. For any pulse width, a typical set of Brillouin gain spectrum is chosen. The fitting and calculation results by the Lorentzian, Gaussian, pseudo-Voigt and Voigt profile-based algorithms are shown diagrammatically in Figure 7. The extracted *g*_BM_, *v*_B_, Δ*v*_B_, and *E* are included in Table 7. *g*_BMM_, *v*_BM_ and Δ*v*_BM_ are the mean values of the extracted *g*_BM_, *v*_B_ and Δ*v*_B_, respectively. From Figure 7 it can be seen that there is a considerable difference between the curves fitted by the Lorentzian, Gaussian profile-based algorithms and the measured one whatever the pulse width is employed. Therefore, the parameters extracted by the Lorentzian, Gaussian profile-based algorithms in Table 7 may contain errors. The difference between the curves fitted by the Lorentzian increases with decreasing pulse width and at the same time, the difference between the curves fitted by the Gaussian increases with increasing pulse width which is consistent with [32].

According to Table 7, the values of *v*_B_ extracted by different models are quite similar. If the pulse width is 10 ns, the mean values of the differences in the extracted Brillouin frequency shift between the Lorentzian, Gaussian, pseudo-Voigt profile-based algorithms and the Voigt profile-based algorithm are only 0.07 MHz, 0.02 MHz and 0.01 MHz, respectively. 

The other cases have similar results. However, the differences in the extracted Δ*v*_B_ between the Lorentzian, Gaussian profiles and the pseudo-Voigt, Voigt profiles are remarkable. There is a very good agreement between the curves fitted by the pseudo-Voigt, Voigt profile-based algorithms and the measured ones. Therefore, Δ*v*_B_ extracted by the Lorentzian and Gaussian profile-based algorithms has significant errors. The sum squared errors *E* of the Lorentzian and Gaussian profile-based algorithms are much larger than that of the pseudo-Voigt and Voigt profile-based algorithms. However, in theory, the parameters extracted by the pseudo-Voigt profile-based algorithm may contain errors (Table 1). From the accuracy point of view, the Voigt model is the best one. However, the computation time of the Voigt profile-based algorithm is much more than that of the Lorentzian, Gaussian and pseudo-Voigt profile-based algorithms. The Voigt profile-based algorithm suffers from the weakness of low arithmetic efficiency. Therefore, the computation time is the key factor of the algorithm.

**Figure 8:**
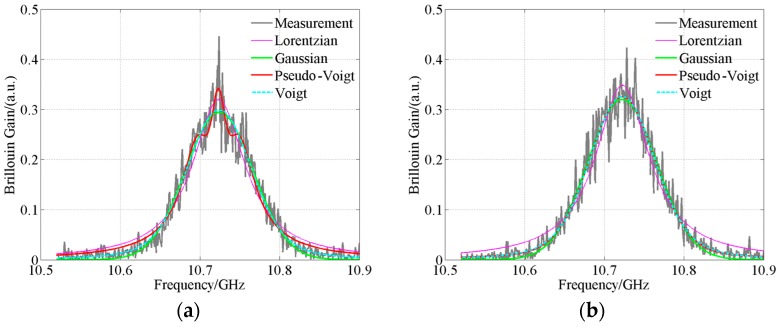
Waveshapes fitted by different models, measured signals with average number of 2^10^. (**a**) Pseudo-Voigt model converges to an erroneous solution; (**b**) Pseudo-Voigt model converges to a correct solution.

In addition to the spectra with high SNR, the key parameters in the spectra with low SNR also need to be extracted. The average number of waveforms is set to 2^10^ and the pulse width is set to 10 ns. The other parameters are the same as the high SNR ones. 8501 sets of Brillouin gain spectra are acquired by AV6419. The mean SNR of the measured spectra is 15.18 dB. The fitting and calculation results by the four models are shown in Figure 8. The statistical results of *g*_BM_, *v*_B_, Δ*v*_B_ and *E* extracted by different models are summarized in Table 8 and the statistical results of the extracted model parameters [14,17,22,33] are presented in Table 9. 

According to Table 8 and Table 9, the values of *v*_B_ extracted by different models are quite similar. The mean values of the differences in the extracted Brillouin frequency shift between the Lorentzian, Gaussian, pseudo-Voigt profile-based algorithms and the Voigt profile-based algorithm are only 0.32 MHz, 0.17 MHz and 0.17 MHz, respectively. However, there is varying degrees difference in *E* and the extracted *g*_BM_, Δ*v*_B_ between different models. From Section 5.1, the Voigt profile-based algorithm can extract parameters with the highest accuracy. Therefore, the other profile-based algorithms will introduce more significant errors. In Figure 8a, the pseudo-Voigt profile-based algorithm converges to an erroneous solution. Therefore, the significant errors are inevitable in the extracted parameters. The results for the noisy measured spectra (Figure 8 and Table 8 and Table 9) are similar to the results for the measured spectra with high SNR (Figure 7 and Table 7). The results further validate the high accuracy of the Voigt profile-based algorithm. The errors in *g*_BM_ extracted by the pseudo-Voigt profile-based algorithm in Table 8 are significant. However, the corresponding sum of the squared normal distances *E* is not so large. This is due to the fact that *E* is calculated based on discrete spectra signal. Significant errors in *g*_BG_ and *g*_BL_ do not necessarily correspond to significant error in *E*. *g*_BM_ is calculated by:

*g*_BM_*= g*_BG_ + *g*_BL_ (19)

It means that the error in *g*_BM_ depends solely on the errors in *g*_BG_ and *g*_BL_.

The above results for measured spectra are consistent with the results for numerically generated spectra in Section 5.1.

#### 5.2.2. Comparison of different algorithms based on Voigt profile

**Figure 9:**
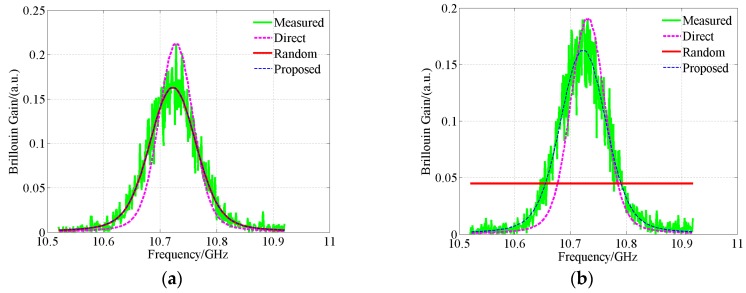
The measured spectra and the waveshapes fitted by three Voigt profile-based algorithms, measured signals with average number of 2^10^. (**a**) Random algorithm converges; (**b**) Random algorithm diverges.

The same 8501 sets of Brillouin gain spectra with low SNR are used in this part. The statistical results of errors in the extracted key parameters, the computation time, the iteration number and the sum of the squared normal distances corresponding to the direct algorithm, the random algorithm and the proposed algorithm are presented in Table 10. The measured spectra and the waveshapes fitted by the three Voigt profile-based algorithms are shown in Figure 9.

The proposed algorithm can converge in all cases and the convergence rate of the random algorithm is 84.08%. Once diverged, the error in the parameters extracted by the random algorithm is quite large, which will hide the accuracy of the converged cases. Therefore, for the random algorithm, only the errors corresponding to the convergence cases are included in Table 10. For the direct algorithm, the extracted parameters for six sets of spectra are complex numbers and they are excluded in Table 10. According to the previous investigations, the proposed algorithm can converge to the optimal solution in all cases. Therefore, the extracted parameters can be taken as the exact values. The corresponding maximum differences in the extracted parameters *g*_M_, *v*_B_ and Δ*v*_B_ between the proposed algorithm and the other two algorithms are represented by *E_g_*_max_, *E_v_*_max_ and *E*_Δ*v*max_, respectively. It is noted in Table 10 that the parameters extracted by the random algorithm and the direct algorithm have significant errors even if the divergence cases have been eliminated. If the temperature coefficient is 1.29 MHz/°C, the mean errors in the temperature measured by the two algorithms are 6.57 °C and 6.91 °C, respectively. The maximum errors in the temperature measurement respectively are 26.95 °C and 639.78 °C. Not only the accuracy, but the computation time of the random algorithm is also more than that of the proposed algorithm. The computation time and the sum squared error of the random algorithm are about one and two times larger than that of the proposed algorithm even for the converged cases.

To sum up, the proposed algorithm can converge to the optimal solution in all cases, regardless of the numerically generated or measured signals, the values of pulse width and SNR used. The parameters can be extracted by the proposed algorithm with the highest accuracy. The proposed algorithm is a little hard to program. At the same time, the computation time is more than those of the Lorentzian, Gaussian and pseudo-Voigt profile-based algorithms. Certainly, we can reduce the computation time by use of a high-performance computer.

**Figure 10:**
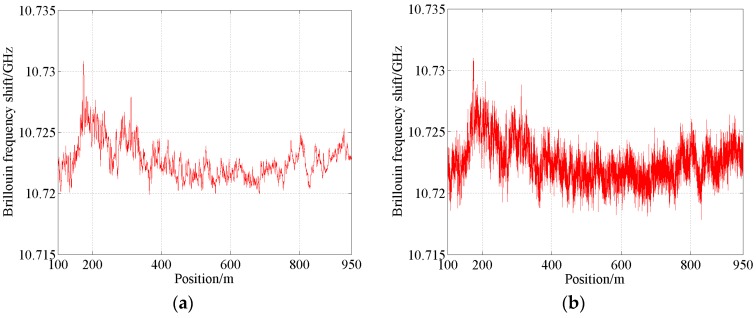
Changes of the extracted Brillouin frequency shift with fiber position. (**a**) Pulse width = 10 ns, average number = 2^18^; (**b**) Pulse width = 10 ns, average number = 2^10^.

Although the fiber is not deployed in real equipment or the real civil infrastructure and at the same time, the spectra are measured at ambient temperature, the wrapped fiber is subject to strain. Changes of the extracted Brillouin frequency shift with fiber position for the measured spectra in Figure 7a and Figure 8 are provided in Figure 10. The Brillouin frequency shift varies significantly with fiber position. Additionally, the pulse width and SNR are different for the different measured spectra. In fact, the variation of Brillouin frequency shift along the fiber may be a little more complex than that of real conditions. Therefore, it can simulate well the influence caused by the temperature and strain in practical cases. The proposed algorithm has been fully validated by a large number of representative and typical numerically generated spectra and measured spectra. Therefore, it can effectively extract key parameters from measured spectra in practical cases.

## 6. Conclusions

Existing key parameters extraction algorithms for Brillouin gain spectra are mainly based on simplified models, and the extracted parameters may have significant errors. Based on the exact model (Voigt profile), the key parameters extraction for fiber Brillouin distributed sensors is systematically investigated in the paper. The conclusions are as follows:

(1) The parameters extracted by the Lorentzian, Gaussian profile-based algorithms easily contain significant errors at different values of pulse width. The existing pseudo-Voigt profile-based algorithm may introduce significant error for the case with low SNR. The proposed Voigt profile-based algorithm is the most accurate one among all four tested profile-based algorithms.
(2) To improve the real-time performance of fiber Brillouin distributed sensing, a key parameter extraction algorithm for Brillouin gain spectrum based on the exact Voigt profile is proposed. The objective function is presented using the least-squares method. The Levenberg-Marquardt algorithm is used to minimize the objective function and consequently extract the key parameters. The initial values obtainment method and the convergence criterion are simultaneously given. The number of sample points in Gauss-Hermite quadrature is suggested to choose 100. The proposed Voigt profile-based algorithm can converge in all cases. It is more accurate than that of the random algorithm and the direct algorithm. It also requires less computational effort than the direct algorithm.

## Figures and Tables

**Table 1:** Statistical results of relative errors in the key parameters extracted by different model-based algorithms/%.

Models	Lorentzian		Gaussian		Pseudo-Voigt	
Errors	*E*_max_	*E*_mean_	*E*_max_	*E*_mean_	*E*_max_	*E*_mean_
*g*_BM_	7.55	5.95	−4.33	1.03	1.51	0.3
*v*_B_/MHz	0	0	0	0	0	0
Δ*v*_B_/MHz	−14.85	12.93	10.43	2.23	−1.76	0.31

**Table 2:** Statistical results of errors in the extracted key parameters corresponding to different convergence criterions and the corresponding computation times.

Convergence Criterions	*E_v_*_mean_/GHz	*E*_Δ*v*mean_/GHz	*E_g_*_mean_	*E*_M_	*T*_M_/s	*l*_M_
Equation (17)	0	8.08 × 10^−5^	3.01 × 10^−4^	1.36 × 10^−4^	2.70 × 10^−2^	9.74
Equation (18)	0	8.08 × 10^−5^	3.01 × 10^−4^	1.36 × 10^−4^	2.48 × 10^−2^	7.74
*l* = 500	0	8.08 × 10^−5^	3.01 × 10^−4^	1.36 × 10^−4^	1.2 × 10^−1^	3.27 × 10^2^

**Table 3:** Statistical results of errors in the Voigt waveshape numerically generated by Gauss-Hermite quadrature with different numbers of sample points.

*M*	*E*_1min_	*E*_1max_	*E*_1mean_	*E*_1std_	*E*_2min_	*E*_2max_	*E*_2mean_	*E*_2std_
5	4.36 × 10^−11^	8.68 × 10^−1^	1.30 × 10^−1^	1.94 × 10^−1^	5.40 × 10^−12^	1.20 × 10^−1^	2.64 × 10^−2^	3.71 × 10^−2^
10	1.01 × 10^−15^	5.16 × 10^−1^	5.25 × 10^−2^	9.90 × 10^−2^	1.73 × 10^−16^	8.93 × 10^−2^	1.28 × 10^−2^	2.35 × 10^−2^
30	1.00 × 10^−15^	2.62 × 10^−1^	1.31 × 10^−2^	3.65 × 10^−2^	1.63 × 10^−16^	5.36 × 10^−2^	3.39 × 10^−3^	9.27 × 10^−3^
60	9.81 × 10^−16^	1.51 × 10^−1^	4.65 × 10^−3^	1.65 × 10^−2^	1.62 × 10^−16^	3.57 × 10^−2^	1.27 × 10^−3^	4.43 × 10^−3^
100	9.81 × 10^−16^	9.03 × 10^−2^	1.95 × 10^−3^	8.24 × 10^−3^	1.65 × 10^−16^	2.41 × 10^−2^	5.51 × 10^−4^	2.33 × 10^−3^
200	1.01 × 10^−15^	4.21 × 10^−2^	5.06 × 10^−4^	2.80 × 10^−3^	1.69 × 10^−16^	1.17 × 10^−2^	1.47 × 10^−4^	8.12 × 10^−4^
600	1.38 × 10^−15^	7.89 × 10^−3^	3.59 × 10^−5^	3.22 × 10^−4^	2.62 × 10^−16^	1.99 × 10^−3^	9.52 × 10^−6^	8.37 × 10^−5^

**Table 4:** Statistical results of errors in the key parameters extracted by the Voigt profile-based algorithm with different numbers of sample points, noise-free.

*M*	5	10	30	60	100	200	600	1000
*E_v_*_mean_/GHz	0	0	0	0	0	0	2.58 × 10^−17^	0
*E_v_*_std_/GHz	0	0	0	0	0	0	1.84 × 10^−15^	0
*E*_Δ*v*mean_/GHz	2.47 × 10^−3^	1.18 × 10^−3^	3.53 × 10^−4^	1.62 × 10^−4^	8.92 × 10^−5^	3.64 × 10^−5^	5.31 × 10^−6^	1.76 × 10^−15^
*E*_Δ*v*std_/GHz	2.65 × 10^−3^	1.75 × 10^−3^	8.32 × 10^−4^	4.92 × 10^−4^	3.27 × 10^−4^	1.80 × 10^−4^	3.92 × 10^−5^	3.31 × 10^−14^
*E_g_*_mean_	7.53 × 10^−3^	3.93 × 10^−3^	1.27 × 10^−3^	5.82 × 10^−4^	3.12 × 10^−4^	1.18 × 10^−4^	1.73 × 10^−4^	1.50 × 10^−14^
*E_g_*_std_	1.20 × 10^−2^	7.78 × 10^−3^	3.54 × 10^−3^	2.00 × 10^−3^	1.26 × 10^−3^	6.19 × 10^−4^	2.03 × 10^−3^	2.73 × 10^−13^
*T*_M_/s	8.80 × 10^−3^	9.06 × 10^−3^	1.34 × 10^−2^	1.96 × 10^−2^	2.77 × 10^−2^	4.79 × 10^−2^	1.36 × 10^−1^	2.29 × 10^−1^

**Table 5:** Statistical results of errors in the key parameters extracted by the Voigt profile-based algorithm with different numbers of sample points, SNR = 40 dB.

*M*	5	10	30	60	100	200	600	1000
*E_v_*_mean_/GHz	1.12 × 10^−4^	1.11 × 10^−4^	1.11 × 10^−4^	1.11 × 10^−4^	1.11 × 10^−4^	1.11 × 10^−4^	1.11 × 10^−4^	1.11 × 10^−4^
*E_v_*_std_/GHz	1.48 × 10^−4^	1.47 × 10^−4^	1.47 × 10^−4^	1.47 × 10^−4^	1.47 × 10^−4^	1.47 × 10^−4^	1.46 × 10^−4^	1.46 × 10^−4^
*E*_Δ*v*mean_/GHz	2.63 × 10^−3^	1.46 × 10^−3^	7.40 × 10^−4^	5.78 × 10^−4^	5.18 × 10^−4^	4.80 × 10^−4^	4.63 × 10^−4^	4.60 × 10^−4^
*E*_Δ*v*std_/GHz	2.93 × 10^−3^	2.16 × 10^−3^	1.52 × 10^−3^	1.37 × 10^−3^	1.32 × 10^−3^	1.29 × 10^−3^	1.29 × 10^−3^	1.28 × 10^−3^
*E_g_*_mean_	8.95 × 10^−3^	5.63 × 10^−3^	3.32 × 10^−3^	2.76 × 10^−3^	2.56 × 10^−3^	2.45 × 10^−3^	2.70 × 10^−3^	2.41 × 10^−3^
*E_g_*_std_	1.76 × 10^−2^	1.47 × 10^−2^	1.28 × 10^−2^	1.24 × 10^−2^	1.23 × 10^−2^	1.22 × 10^−2^	1.42 × 10^−2^	1.22 × 10^−2^
*T*_M_/s	9.17 × 10^−3^	9.26 × 10^−3^	1.40 × 10^−2^	2.10 × 10^−2^	3.08 × 10^−2^	5.22 × 10^−2^	1.41 × 10^−1^	2.33 × 10^−1^

**Table 6:** Statistical results of errors in the key parameters extracted by different algorithms, numerically generated signals.

Algorithm	Signal Type	*E_v_*_mean_ /GHz	*E_v_*_std_ /GHz	*E*_Δ*v*mean_ /GHz	*E*_Δ*v*max_ /GHz	*E_g_*_mean_	*E_g_*_std_	*T*_M_/s
Direct	Noise-free	2.00 × 10^−3^	2.46 × 10^−3^	2.34 × 10^−4^	1.57 × 10^−4^	1.22 × 10^−2^	1.12 × 10^−1^	7.46 × 10^−3^
	Noisy	8.16 × 10^−3^	1.07 × 10^−2^	7.61 × 10^−3^	8.84 × 10^−3^	5.02 × 10^−2^	6.55 × 10^−2^	6.34 × 10^−3^
Random	Noise-free	2.33 × 10^4^	1.23 × 10^5^	1.90 × 10^4^	2.35 × 10^5^	5.03 × 10^−2^	2.38 × 10^−1^	1.07 × 10^−1^
	Noisy	4.21 × 10^8^	3.55 × 10^9^	2.26 × 10^8^	2.00 × 10^9^	9.04 × 10^−2^	4.73 × 10^−1^	1.24 × 10^−1^
Proposed	Noise-free	0	0	8.92 × 10^−5^	3.27 × 10^−4^	3.12 × 10^−4^	1.26 × 10^−3^	2.77 × 10^−2^
	Noisy	1.12 × 10^−3^	1.49 × 10^−3^	3.90 × 10^−3^	5.30 × 10^−3^	1.88 × 10^−2^	3.22 × 10^−2^	4.85 × 10^−2^

**Table 7:** Mean value of the key parameters, *E* and *T* by different models for spectra with different pulse widths, measured signals with average number of 2^18^.

Models	Pulse Widths	10 ns	20 ns	50 ns	100 ns	200 ns
Lorentzian	*g*_BMM_	2.95 × 10^−1^	9.42 × 10^−1^	2.23	3.66	5.06
	*v*_BM_/GHz	1.07 × 10^1^	1.07 × 10^1^	1.07 × 10^1^	1.07 × 10^1^	1.07 × 10^1^
	Δ*v*_BM_/GHz	8.22 × 10^−2^	4.97 × 10^−2^	3.69 × 10^−2^	3.55 × 10^−2^	3.60 × 10^−2^
	*E*_M_	1.03 × 10^−1^	4.72 × 10^−1^	1.03	2.44	4.57
	*T*_M_/s	2.41 × 10^−3^	2.19 × 10^−3^	1.97 × 10^−3^	1.93 × 10^−3^	1.89 × 10^−3^
Gaussian	*g*_BMM_	2.73 × 10^−1^	8.64 × 10^−1^	2.02	3.32	4.59
	*v*_BM_/GHz	1.07 × 10^1^	1.07 × 10^1^	1.07 × 10^1^	1.07 × 10^1^	1.07 × 10^1^
	Δ*v*_BM_/GHz	1.02 × 10^−1^	6.22 × 10^−2^	4.66 × 10^−2^	4.48 × 10^−2^	4.54 × 10^−2^
	*E*_M_	8.23 × 10^−3^	1.14 × 10^−1^	1.1	3.1	6.3
	*T*_M_/s	1.74 × 10^−3^	1.83 × 10^−3^	2.22 × 10^−3^	2.24 × 10^−3^	2.23 × 10^−3^
Pseudo-Voigt	*g*_BMM_	2.75 × 10^−1^	8.81 × 10^−1^	2.13	3.5	4.84
	*v*_BM_/GHz	1.07 × 10^1^	1.07 × 10^1^	1.07 × 10^1^	1.07 × 10^1^	1.07 × 10^1^
	Δ*v*_BM_/GHz	9.99 × 10^−2^	5.96 × 10^−2^	4.23 × 10^−2^	4.04 × 10^−2^	4.09 × 10^−2^
	*E*_M_	1.61 × 10^−3^	6.56 × 10^−3^	4.48 × 10^−2^	1.25 × 10^−1^	2.66 × 10^−1^
	*T*_M_/s	9.83 × 10^−3^	6.34 × 10^−3^	3.48 × 10^−3^	3.59 × 10^−3^	3.57 × 10^−3^
Voigt	*g*_BMM_	2.74 × 10^−1^	8.82 × 10^−1^	2.11	3.46	4.79
	*v*_BM_/GHz	1.07 × 10^1^	1.07 × 10^1^	1.07 × 10^1^	1.07 × 10^1^	1.07 × 10^1^
	Δ*v*_BM_/GHz	9.86 × 10^−2^	5.93 × 10^−2^	4.26 × 10^−2^	4.07 × 10^−2^	4.12 × 10^−2^
	*E*_M_	3.09 × 10^−3^	5.66 × 10^−3^	3.36 × 10^−2^	9.55 × 10^−2^	1.93 × 10^−1^
	*T*_M_/s	1.33 × 10^−1^	7.24 × 10^−2^	6.54 × 10^−2^	6.46 × 10^−2^	6.27 × 10^−2^

**Table 8:** Statistical results of *g*_BM_, *v*_B_, Δ*v*_B_, *E* and *T* extracted by different models, measured signals with average number of 2^10^.

Models	Parameters	Max	Min	Mean	Std
Lorentzian	*g*_BM_	3.59 × 10^−1^	2.82 × 10^−1^	3.19 × 10^−1^	1.23 × 10^−2^
	*v*_B_/GHz	1.07 × 10^1^	1.07 × 10^1^	1.07 × 10^1^	1.76 × 10^−3^
	Δ*v*_B_/GHz	8.98 × 10^−2^	7.50 × 10^−2^	8.30 × 10^−2^	1.67 × 10^−3^
	*E*	4.46 × 10^−1^	2.16 × 10^−1^	3.12 × 10^−1^	3.20 × 10^−2^
	*T*/s	1.37 × 10^−2^	1.46 × 10^−3^	2.07 × 10^−3^	2.81 × 10^−4^
Gaussian	*g*_BM_	3.31 × 10^−1^	2.62 × 10^−1^	2.95 × 10^−1^	1.11 × 10^−2^
	*v*_B_/GHz	1.07 × 10^1^	1.07 × 10^1^	1.07 × 10^1^	1.66 × 10^−3^
	Δ*v*_B_/GHz	1.09 × 10^−1^	9.55 × 10^−2^	1.02 × 10^−1^	1.61 × 10^−3^
	*E*	3.06 × 10^−1^	1.43 × 10^−1^	2.14 × 10^−1^	2.42 × 10^−2^
	*T*/s	7.91 × 10^−3^	1.41 × 10^−3^	1.91 × 10^−3^	2.18 × 10^−4^
Pseudo-Voigt	*g*_BM_	1.76	1.53 × 10^−1^	2.98 × 10^−1^	1.99 × 10^−2^
	*v*_B_/GHz	1.07 × 10^1^	1.07 × 10^1^	1.07 × 10^1^	1.67 × 10^−3^
	Δ*v*_B_/GHz	1.08 × 10^−1^	7.39 × 10^−2^	9.98 × 10^−2^	2.52 × 10^−3^
	*E*	3.62 × 10^−1^	1.32 × 10^−1^	2.03 × 10^−1^	2.52 × 10^−2^
	*T*/s	8.87 × 10^−2^	2.98 × 10^−3^	8.51 × 10^−3^	3.57 × 10^−3^
Voigt	*g*_BM_	3.34 × 10^−1^	2.50 × 10^−1^	2.95 × 10^−1^	1.23 × 10^−2^
	*v*_B_/GHz	1.07 × 10^1^	1.07 × 10^1^	1.07 × 10^1^	1.70 × 10^−3^
	Δ*v*_B_/GHz	1.06 × 10^−1^	8.97 × 10^−2^	9.85 × 10^−2^	1.95 × 10^−3^
	*E*	2.97 × 10^−1^	1.34 × 10^−1^	2.03 × 10^−1^	2.39 × 10^−2^
	*T*/s	3.82	3.79 × 10^−2^	2.52 × 10^−1^	3.83 × 10^−1^

**Table 9:** Statistical results of model parameters extracted by different models, measured signals with average number of 2^10^.

Models	Parameters	Max	Min	Mean	Std
Lorentzian	*g*_BM_	3.59 × 10^−1^	2.82 × 10^−1^	3.19 × 10^−1^	1.23 × 10^−2^
	*v*_B_/GHz	1.07 × 10^1^	1.07 × 10^1^	1.07 × 10^1^	1.76 × 10^−3^
	Δ*v*_B_/GHz	8.98 × 10^−2^	7.50 × 10^−2^	8.30 × 10^−2^	1.67 × 10^−3^
Gaussian	*g*_BM_	3.31 × 10^−1^	2.62 × 10^−1^	2.95 × 10^−1^	1.11 × 10^−2^
	*v*_B_/GHz	1.07 × 10^1^	1.07 × 10^1^	1.07 × 10^1^	1.66 × 10^−3^
	Δ*v*_B_/GHz	1.09 × 10^−1^	9.55 × 10^−2^	1.02 × 10^−1^	1.61 × 10^−3^
Pseudo-Voigt	*g*_BL_	1.12	−1.37 × 10^−1^	3.59 × 10^−2^	1.22 × 10^−1^
	*v*_B_/GHz	1.07 × 10^1^	1.07 × 10^1^	1.07 × 10^1^	1.67 × 10^−3^
	Δ*v*_BL_/GHz	2.23 × 10^7^	1.46 × 10^−4^	5.36 × 10^5^	1.19 × 10^6^
	*g*_BG_	1.44	−7.96 × 10^−1^	2.62 × 10^−1^	1.22 × 10^−1^
	Δ*v*_BG_/GHz	2.70 × 10^−1^	2.04 × 10^−6^	9.72 × 10^−2^	1.44 × 10^−2^
Voigt	*A*	3.86 × 10^−2^	3.09 × 10^−2^	3.46 × 10^−2^	1.33 × 10^−3^
	*v*_B_/GHz	1.07 × 10^1^	1.07 × 10^1^	1.07 × 10^1^	1.70 × 10^−3^
	Δ*v*_BL_/GHz	3.58 × 10^−2^	1.27 × 10^−2^	2.23 × 10^−2^	3.79 × 10^−3^
	Δ*v*_BG_/GHz	9.92 × 10^−2^	7.04 × 10^−2^	8.68 × 10^−2^	3.66 × 10^−3^

**Table 10:** Statistical results of errors in the extracted key parameters, computation time, iteration number and sum of the squared normal distances corresponding to three Voigt profile-based algorithms, measured signals with average number of 2^10^.

Algorithms	*E_g_*_mean_	*E_g_*_max_	*E_v_*_mean_/GHz	*E_v_*_max_/GHz	*E*_Δ*v*mean_/GHz	*E*_Δ*v*max_/GHz	*l*_M_	*T*_M_/s	*E*_M_
Direct	4.31 × 10^−2^	1.02 × 10^−1^	8.48 × 10^−3^	3.48 × 10^−2^	2.62 × 10^−2^	8.38 × 10^−2^	-	6.36 × 10^−3^	2.37 × 10^−1^
Random, convergence	8.67 × 10^−3^	1.28 × 10^−1^	8.91 × 10^−3^	8.25 × 10^−1^	6.07 × 10^−2^	4.5	1.55 × 10^2^	8.12 × 10^−1^	1.25 × 10^−1^
Proposed	-	-	-	-	-	-	4.06 × 10^1^	2.77 × 10^−1^	5.09 × 10^−2^
